# Evaluation of Far Ultraviolet-C Light for Decontamination of Organisms in Whole Milk and Chicken Manure

**DOI:** 10.20411/pai.v10i2.801

**Published:** 2025-05-19

**Authors:** Samir Memic, Jennifer L. Cadnum, Curtis J. Donskey

**Affiliations:** 1 Department of Systems Biology, Case Western Reserve University School of Medicine, Cleveland, Ohio; 2 Research Service, Louis Stokes Cleveland VA Medical Center, Cleveland, Ohio; 3 Department of Medicine, Case Western Reserve University School of Medicine, Cleveland, Ohio; 4 Geriatric Research, Education, and Clinical Center, Louis Stokes Cleveland VA Medical Center, Cleveland, Ohio

**Keywords:** Far Ultraviolet-C, Dairy, Highly Pathogenic Avian Influenza, Chickens

## Abstract

**Background:**

The dissemination of highly pathogenic avian influenza (HPAI) A(H5N1) in US poultry and dairy cows poses a public health threat. Farm workers caring for infected animals are at risk to acquire infections due to exposure to contaminated milk or poultry feces and secretions. Far ultraviolet-C (UV-C) light could provide continuous decontamination of surfaces and air in agricultural settings, but efficacy against organisms in whole milk or chicken manure is unclear.

**Methods:**

We examined the efficacy of far UV-C light against bacteriophage MS2 and methicillin-resistant *Staphylococcus aureus* (MRSA) in phosphate-buffered saline (PBS), 5% fetal calf serum, whole milk, or 5%, 10%, and 25% chicken manure, both in liquid suspension and dried on surfaces. We also compared the efficacy of 300 mJ/cm^2^ doses of far UV-C and 254-nm UV-C light against the test organisms in liquid droplets or droplets dried on surfaces.

**Results:**

For both test organisms, far UV-C achieved significantly smaller log_10_ reductions in whole milk and in chicken manure suspensions than in PBS or 5% fetal calf serum, both in liquid suspension and when dried on surfaces (*P*<0.0001). In whole milk, average reductions of both organisms with all doses were ≤1.2 log_10_ in liquid suspensions and ≤2.4 log_10_ when dried on surfaces. We found 254-nm UV-C was significantly more effective in reducing MRSA and MS2 dried on surfaces in whole milk or in 10% chicken manure (*P*≤0.02) but not in liquid droplets (*P*>0.05) except 5% chicken manure (*P*<0.001).

**Conclusions:**

Our results suggest that in the absence of prior cleaning and disinfection far UV-C and 254-nm UV-C light technologies may have limited efficacy as an adjunctive method to reduce the risk for transmission of HPAI from surfaces in high-risk settings on farms.

## INTRODUCTION

The dissemination of highly pathogenic avian influenza (HPAI) A(H5N1) in US poultry and dairy cows poses a public health threat [1, 2]. Farm workers caring for infected animals are at risk to acquire infections [1–3]. Milk from infected dairy cows contains high concentrations of infectious virus that dairy workers may be exposed to while milking [4]. Contamination of milking equipment may also contribute to spread from infected to susceptible cows [4]. Contaminated poultry feces and secretions pose a similar transmission risk [2]. Personal protective equipment (PPE) is recommended for workers at risk for exposure to influenza A(H5N1), but adherence is suboptimal, in part due to the acceptability of the recommended PPE under working conditions on farms [5].

Influenza viruses remain infectious for hours in milk, on milking equipment, and in poultry manure [4, 6]. Technologies that provide continuous decontamination of surfaces and air could be a useful adjunct to current prevention measures. Far ultraviolet-C (UV-C) light (222 nm) is a candidate technology because it can be operated while people are present at doses within proposed threshold limit values [7]. UV-C light is effective against a wide range of pathogens, including influenza viruses [7–10]. However, the efficacy of UV-C light may be substantially reduced due to limited penetration of opaque liquids and/or by high levels of organic material present in milk and poultry manure [11–13]. Previous studies have demonstrated that the efficacy of 254-nm UV-C light against organisms in milk can be significantly enhanced using strategies that increase exposure of the organisms to UV-C (eg, turbulent and thin film flow) [11, 12]. We are not aware of similar studies with far UV-C.

During milking, workers may be exposed to equipment contaminated with liquid milk droplets or milk droplets that have dried on surfaces. If far UV-C light is substantially reduced in milk droplets or in milk dried on surfaces, it may have limited value for surface disinfection unless applied after manual cleaning and disinfection. Here, we tested the efficacy of a far UV-C technology against organisms in whole milk and suspensions of chicken manure, including in droplets on surfaces and dried on surfaces.

## METHODS

### Description of the Far UV-C Light Technology

A wall-mounted far UV-C technology (Pathogen Suppression System, Mynatek, Inc.) was used [9, 10]. The device uses 3 krypton-chloride excimer lamps that emit a primary wavelength of 222 nm with filters to block emitted wavelengths >230 nm. Each device contains 3 lamps with a field of illumination of 60° per lamp. For this study, the devices were mounted on posts. The device includes proprietary sensors that detect people within the field of illumination, including individuals remaining motionless. The technology contains sensors that detect people and reduce output as needed to maintain dosing within 8-hour threshold limit values proposed for far UV-C (161 mJ/cm^2^ for eyes and 479 mJ/cm^2^ for skin) [7, 9, 10]. Alternatively, the technology can be programmed to deliver lower doses that are within threshold limit values without the requirement for the use of sensors to adjust the dose.

### Test Protocol and Organisms

The study protocol was approved by the Research and Development and Biosafety Committees at the Louis Stokes Cleveland VA Medical Center. Research personnel were not exposed to far UV-C light as the devices were only operated when personnel were outside the area of exposure. For an area such as a milking parlor, it is anticipated that devices would be placed 2 to 3 meters from the work area adjacent to each cow. With 2 devices placed 6 meters apart at a height of 2 meters, irradiance measured with a radiometer (UIT2400 Handheld Light Meter for 222 nm, Ushio America) at 3 meters from each device at a height of 1 meter was 16.0 µW/cm^2^, resulting in a dose of 57.6 mJ/cm^2^ after 1 hour of continuous operation.

The test organisms included a clinical methicillin-resistant *Staphylococcus aureus* (MRSA) isolate and bacteriophage MS2 (American Type Culture Collection 15597-B1). *S. aureus* is a common cause of mastitis and bacteriophage MS2 is commonly used as a surrogate for viral respiratory pathogens [7]. Bacteriophage MS2 is a non-enveloped virus that is relatively resistant to killing by UV-C light in comparison to enveloped viruses [14]. Bacteriophage MS2 was propagated in *Escherichia coli* 15597 [15].

The organisms were suspended in phosphate-buffered saline (PBS), 5% fetal calf serum, commercial whole milk, or Expert Gardener Organics Chicken Manure All-Natural Plant Food (Scotts) diluted to 5%, 10%, and 25% in tap water. Five mL suspensions containing 10^4^-10^5^ colony-forming units (CFU) or plaque-forming units (PFU) per mL of MRSA or bacteriophage MS2, respectively, in petri dishes 100 cm from the device were exposed to a total dose of 300 mJ/cm^2^ of far UV-C. A radiometer for 222 nm far UV-C light was used to measure irradiance at the site of the samples in order to calculate far UV-C doses. The suspensions were stirred at 200 rpm to increase mixing and the potential for UV-C light exposure. At multiple far UV-C doses ranging from 25 to 300 mJ/cm^2^, aliquots were collected to quantify viable MRSA or bacteriophage MS2. Control suspensions of the test organisms that were not exposed to far UV-C were sampled at the same time points.

For MRSA quantification, aliquots of the suspensions were serially diluted in PBS and plated on CHROMagar *Staph aureus* with 6 μg/mL cefoxitin for MRSA [10]. For bacteriophage MS2 quantification, the double-layer agar technique was used with *E. coli* ATCC 15597 as the host bacterium [7, 15]. To evaluate efficacy against organisms dried on surfaces, 10 µL aliquots of the suspensions were spread to cover 20 mm diameter steel disks and allowed to air dry before exposure to far UV-C. The disks were processed to quantify organisms as previously described [7, 9]. The limit of detection was ~1 log_10_ for both MRSA and bacteriophage MS2. Experiments were repeated 3 times. Results were graphed as mean log_10_ reductions ± standard error.

### Evaluation of Standard 254-nm UV-C Light

Far UV-C light could potentially be less effective than standard 254-nm UV-C light in reducing organisms associated with a soil load because it is highly absorbed by proteins and other organic material [7]. Therefore, we compared the efficacy of a 300 mJ/cm^2^ dose of far UV-C vs the same dose of 254-nm UV-C light. The 254-nm UV-C light was delivered using a CleanSlate Vital Solution device (CleanSlate UV, LLC) with a 20-second exposure using the same soil suspensions. Ten µL aliquots were dried on steel disks or pipetted onto the disks without spreading and tested as a liquid droplet. The testing was completed with liquid droplets rather than 5 mL suspensions due to technical reasons (ie, the CleanSlate box device does not accommodate the larger test set-up) and because it is likely that many real-world exposures might involve droplets rather than larger volumes. Controls for the test organisms that were handled identically but not exposed to 254-nm or far UV-C were sampled at baseline and at the same time the post-exposure samples. Experiments were repeated 3 times. Results were graphed as mean log_10_ reductions + standard error.

### Data Analysis

For all experiments, generalized additive modeling was performed to compare overall reductions among the groups with a Bonferroni correction for multiple comparisons. Mean log_10_ reductions achieved with each of the soil loads were compared with the reduction achieved in PBS. Data were analyzed using R version 3.5.0 software (The R Foundation for Statistical Computing, Vienna, Austria).

## RESULTS

**Figure 1.**
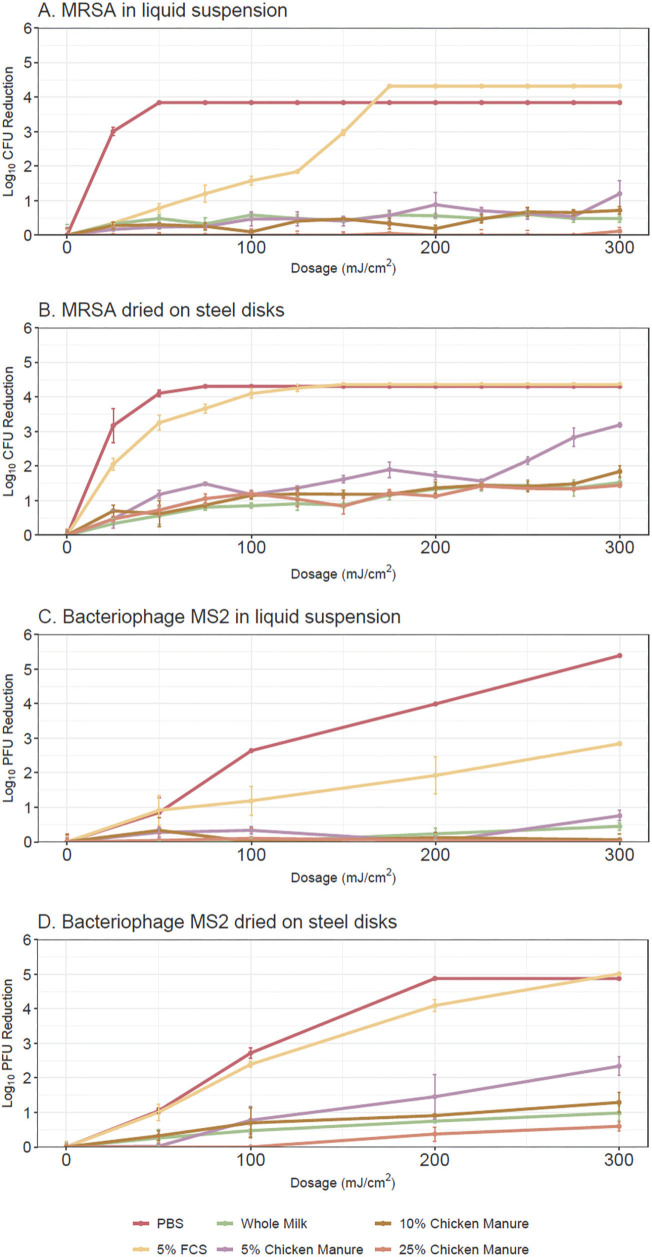
**Far ultraviolet-C light dose-response curves for methicillin-resistant *Staphylococcus aureus* (MRSA) in liquid suspensions (A) and suspensions dried on steel disks (B) and for bacteriophage MS2 in liquid suspensions (C) and suspensions dried on steel disks (D).** Organisms were suspended in phosphate-buffered saline, 5% fetal calf serum, commercial whole milk, or chicken manure diluted to 5%, 10%, and 25% in tap water. Error bars represent standard error. CFU, colony-forming unit; PFU, plaque-forming unit; FCS, fetal calf serum; PBS, phosphate-buffered saline.

Figure 1 shows the far UV-C dose-response curves for MRSA and bacteriophage MS2 in liquid suspension and for suspensions dried on steel disks. In control suspensions not exposed to far UVC, the concentration of MRSA and bacteriophage MS2 remained stable at 10^4^-10^5^ CFU or PFU per mL, respectively. The generalized additive models explained 95.1% (R2=0.947) and 91.5% of the deviance (R2=0.905) for MRSA and bacteriophage MS2, respectively, with significant contributions from both parametric coefficients and smooth terms. In PBS, MRSA was reduced to undetectable levels after a dose of 50 mJ/cm^2^, whereas doses of 200 to 300 mJ/cm^2^ were required to reduce bacteriophage MS to undetectable levels. In 5% fetal calf serum, reductions of both organisms were reduced in liquid suspension compared to PBS, but not when dried on steel disks. For both organisms, reductions achieved by far UV-C were significantly lower in whole milk and diluted chicken manure versus in PBS or 5% fetal calf serum, both in liquid suspension and when dried on surfaces (*P*<0.0001). In whole milk, average reductions of both organisms with all doses were ≤1.2 log_10_ in liquid suspensions and ≤2.4 log_10_ when dried on surfaces. At the 300 mJ/cm^2^ dose, the average reductions of MRSA and bacteriophage MS2 in whole milk in liquid suspension were 1.2 and 0.7 log_10_, respectively, and in whole milk dried on steel disks reductions were 3.1 and 2.4 log_10_, respectively.

**Figure 2.**
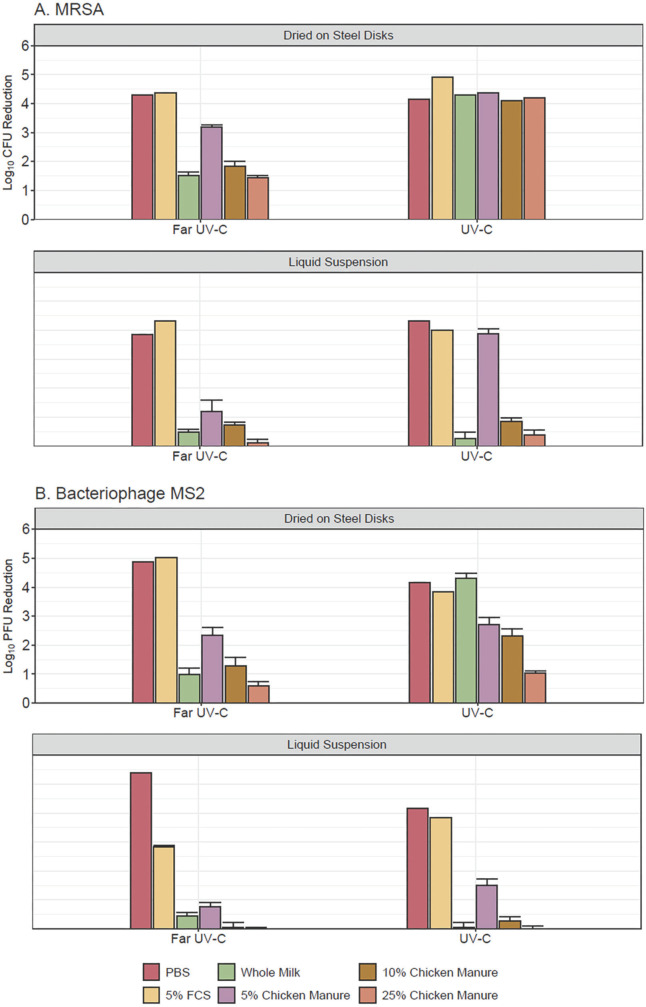
**Efficacy of 300 mJ/cm^2^ doses of far ultraviolet-C (UV-C) and 254-nm UV-C light in reducing methicillin-resistant *Staphylococcus aureus* (MRSA) (A) dried on steel disks and in liquid suspension and bacteriophage MS2 (B) dried on steel disks or in liquid suspension.** Organisms were suspended in phosphate-buffered saline, 5% fetal calf serum, commercial whole milk, or chicken manure diluted to 5%, 10%, and 25% in tap water. Error bars represent standard error. CFU, colony-forming units; PFU, plaque-forming units; FCS, fetal calf serum; PBS, phosphate-buffered saline.

Figure 2 shows the efficacy of 300 mJ/cm^2^ doses of far UV-C and 254-nm UV-C light in reducing MRSA and bacteriophage MS2 in liquid suspension droplets or in droplets dried on steel disks. Both far UV-C and 254-nm UV-C reduced MRSA in PBS and 5% fetal calf serum by ≥3.8 log_10_ and bacteriophage MS2 by ≥2.8 log_10_. The 254-nm UV-C was not significantly more effective in reducing MRSA and MS2 in liquid suspensions (*P*>0.05) except 5% chicken stool (*P*<0.001). However, 254-nm UV-C was significantly more effective in reducing MRSA and MS2 dried on surfaces in whole milk or diluted chicken manure (except MS2 in 5% or 25% chicken manure) (*P*≤0.02).

## DISCUSSION

There is an urgent need for development of effective strategies to reduce the risk for spread of HPAI A(H5N1) on farms. UV-C light is a promising technology because it is highly effective against influenza viruses, and far UV-C technologies could potentially be used safely while farm workers are present. We demonstrated that far UV-C light was effective against MRSA and bacteriophage MS2 in PBS or 5% fetal calf serum. However, whole milk and chicken manure markedly reduced the efficacy of far UV-C against MRSA and bacteriophage MS2 in a stirred liquid suspension, in 10 µL droplets, and when dried on surfaces. These results suggest that far UV-C light technologies may have limited efficacy as a standalone adjunctive method to reduce the risk for transmission of HPAI in high-risk settings on farms. These technologies could potentially be useful as an adjunct after completion of standard cleaning and disinfection has been performed to eliminate organic material.

Far UV-C light is strongly absorbed by proteins and other biomolecules [16]. This feature of far UV-C light is advantageous because exposure to doses within proposed threshold limit values may be safe [7, 16]. Far UV-C light does not substantially penetrate the dead cell layer of skin (stratum corneum) and does not substantially penetrate through corneal epithelium of the eye, thereby preventing exposure of germinative cells within the eye [15]. However, absorption of far UV-C may also reduce efficacy if organisms are associated with organic material. Efficacy of far UV-C was only modestly reduced in the presence of 5% fetal calf serum but was markedly reduced by whole milk and chicken manure. These findings suggest that the relatively high concentrations of protein and fat in whole milk may reduce efficacy of far UV-C light [13].

The efficacy of 254-nm UV-C was similarly reduced in droplets of whole milk and diluted chicken manure. However, 254-nm UV-C was more effective than far UV-C in reducing MRSA and MS2 when dried on surfaces in whole milk or 10% chicken manure. The increased efficacy of 254-nm UV-C against the organisms in this condition could potentially be related to less absorption by proteins and other biomolecules [16]. Alternatively, we cannot exclude the possibility that the different delivery method (ie, 20-second high-intensity exposure) might have resulted in greater efficacy.

Our study has some limitations. Testing was conducted in a laboratory setting. It is not clear how well the test conditions (ie, stirred suspension, 10 µL droplet, and droplet dried on surfaces) mimic real-world exposures to infected milk or chicken manure. Only 1 bacterium and 1 virus were tested. The enveloped influenza virus is more susceptible to UV-C light than the non-enveloped surrogate virus bacteriophage MS2 [8, 14]. Therefore, it is possible that our findings underestimate the potential impact of far UV-C in reducing HPAI. We did not evaluate efficacy against aerosolized viral particles that could potentially contribute to transmission in agricultural settings. A chicken manure plant food product was used that may differ from chicken manure on farms.

In summary, far UV-C light was effective in reducing MRSA and bacteriophage MS2 in PBS and 5% fetal calf serum. However, whole milk and chicken manure markedly reduced the efficacy of far UV-C light, both in liquid suspension and when dried on surfaces. These results suggest that far UV-C light technologies may have limited usefulness as a standalone method to reduce the risk for transmission of HPAI from surfaces in agricultural settings. Additional work is needed to determine if far UV-C could be useful to reduce airborne viral particles in farm settings or as an adjunctive measure to reduce surface contamination after completion of manual cleaning.
